# One-Step Electrochemical Synthesis and Surface Reconstruction of NiCoP as an Electrocatalyst for Bifunctional Water Splitting

**DOI:** 10.3390/ma16041529

**Published:** 2023-02-11

**Authors:** Minhao Sheng, Yawei Yang, Xiaoqing Bin, Wenxiu Que

**Affiliations:** Electronic Materials Research Laboratory, International Center for Dielectric Research, Shaanxi Engineering Research Center of Advanced Energy Materials and Devices, School of Electronic Science and Engineering, Xi’an Jiaotong University, Xi’an 710049, China

**Keywords:** electrocatalyst, electrodeposition, NiCoP, water electrolysis, surface reconstruction

## Abstract

We adopted a simple one-step electrochemical deposition to acquire an efficient nickel cobalt phosphorus (NiCoP) catalyst, which avoided the high temperature phosphatization engineering involved in the traditional synthesis method. The effects of electrolyte composition and deposition time on electrocatalytic performance were studied systematically. The as-prepared NiCoP achieved the lowest overpotential (η_10_ = 111 mV in the acidic condition and η_10_ = 120 mV in the alkaline condition) for the hydrogen evolution reaction (HER). Under 1 M KOH conditions, optimal oxygen evolution reaction (OER) activity (η_10_ = 276 mV) was also observed. Furthermore, the bifunctional NiCoP catalyst enabled a high-efficiency overall water-splitting by applying an external potential of 1.69 V. The surface valence and structural evolution of NiCoP samples with slowly decaying stability under alkaline conditions are revealed by XPS. The NiCoP is reconstructed into the Ni(Co)(OH)_2_ (for HER) and Ni(Co)OOH (for OER) on the surface with P element loss, acting as real “active sites”.

## 1. Introduction

Since the policy of “carbon peak and carbon neutrality” was advocated, energy conservation and emission reduction have become the consensus of all mankind. The development of green energy technology is extremely critical to reduce carbon dioxide emissions [1]. The preparation of green hydrogen occupies an important strategic position in the future. Electrolysis of water to hydrogen has the potential to transfer a large amount of renewable energy electricity to the decarbonized green hydrogen industrial sector [2,3].

Conventional water electrolysis reactions involve two electrochemical semi-reactions at the anode and cathode, in which electrochemical kinetics are controlled by HER and OER, respectively. The theoretical decomposition voltage of electrolytic water is 1.23 V. Nevertheless, in practical electrolytic cells, higher energy supply is inevitably required owing to the slow kinetic limiting step of HER and OER [4]. Therefore, the development of various highly active electrolytic water catalysts to reduce the reaction activation energy can significantly reduce the cost of hydrogen production.

In terms of catalytic activity, the commercial market tends to prefer mature noble metal catalysts (Pt, Ir, and Ru), but their high cost, limited reserves, and easy aggregation characteristics urge the development of non-noble metal catalysts [5,6,7,8]. Transition-metal-based electrocatalysts have shown great potential for overall water splitting over the past few years. Various oxides (MoO_x_) [9], hydroxides (Ni(OH)_2_) [10,11], sulfides (MoS_2_) [12], selenides (MoSe_2_) [13], nitrides (VN) [14], and carbides (MXene) [15,16] materials have been extensively reported as water splitting electrocatalysts. Among the aforementioned catalysts, transition metal phosphides (TMPs) possess a hydrogenase-like structure, and are regarded as promising nonprecious electrocatalysts for overall water splitting [17,18,19,20,21,22]. Single metal phosphides have been widely reported in the field of water electrolysis [23]. Liu et al. [24] employed NiS_2_ single-crystal octahedrons as a precursor and designed a 6 nm thin-wall hollow metallic Ni_2_P and metalloid NiP_2_ polymorph through phosphorization. Hierarchically porous W-doped CoP nanoflake arrays on carbon cloth (W-CoP NAs/CC) were synthesized for pH-universal HER [25]. In addition, bimetallic phosphides can improve the electronic states of single metal and highly extend catalytic performance as a result of a synergetic effect. Lin et al. [19] proposed a novel in situ doping-induced lattice strain strategy to synthesize NiCoP/S nanocrystals (NCs), which significantly improved HER performance in a wide pH range. Ye et al. [20] constructed porous NiCoP heterostructures on Ni foam using the hydrothermal and phosphating method. However, the present phosphide preparation process almost always involves high temperature phosphating aftertreatment of transition metal species. The complexity of the multi-step process and the dangerous volatile PH_3_ gas hinder the reproducibility and green friendliness of large-scale catalyst preparation [26,27].

Based on the above considerations, we synthesized highly active NiCoP microspheres on a self-supported conductive carbon cloth by employing a simple one-step electrochemical deposition method. Electrochemical deposition is an effective strategy for catalyst synthesis because of its advantages of being green, safe, controllable, and efficient. Compared with the post-treatment high-temperature phosphating process, we can directly obtain NiCoP active substances by skipping this tedious step. We systematically evaluated the differences between the catalytic properties of single metal (NiP and CoP) and double transition metal phosphide (NiCoP), and optimized the electrochemical deposition time of NiCoP synthesis. Through compositional optimization, NiCoP achieved the lowest overpotential (η_10_ = 111 mV in the acidic condition and η_10_ = 120 mV in the alkaline condition) for HER. Under alkaline conditions, excellent OER activity (η_10_ = 276 mV) was also observed. The overall water splitting device composed of NiCoP/NiCoP was assembled with an initial cell voltage of 1.69 V to achieve a current density of 10 mA cm^−2^ in 1.0 M KOH. Ex situ XPS characterization captured the dynamic structure evolution and surface remodeling during water electrolysis catalysis, indicating that the dissolution of P from the surface of NiCoP to form hydroxide (HER) and oxyhydroxide (OER) remodelers slowly inhibited the electrolysis reaction. Based on these investigations, we believe that rapid electrochemical synthesis of low-cost NiCoP is expected to show potential in the field of water splitting. In addition, our insight into the structural evolution of phosphide after prolonged electrolysis helps to reveal the true active site.

## 2. Methodology

**Figure 1:**
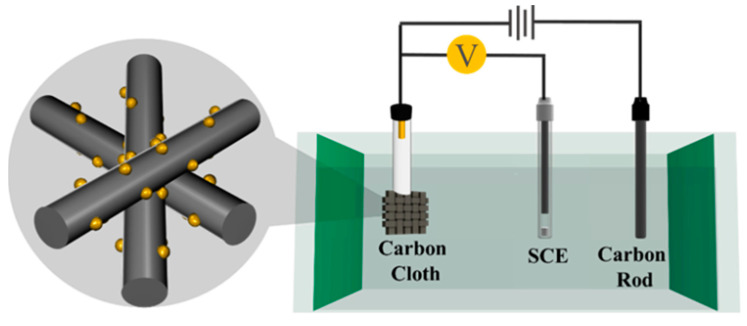
Electrodeposition synthesis procedure of NiCoP.

The carbon cloth (CC) was pretreated with acetone, ethyl alcohol, and ultrapure water; subjected to ultrasonic treatment for 20 min; and dried in an oven at 60 °C for 24 h. As shown in Figure 1, the NiCoP catalyst was obtained by one-step electrochemical deposition at room temperature without any heat-treatment steps. Specifically, the configuration scheme of the precursor solution was as follows: 2 mM CoCl_2_∙6H_2_O, 2 mM NiCl_2_∙6H_2_O, and 10 mM NaH_2_PO_2_∙H_2_O were dissolved in 20 mL of water. The evenly mixed solution was transferred to an electrolytic cell. A three-electrode system was used for electrochemical deposition. The working electrode was carbon cloth (1 cm × 1 cm size), with a carbon rod as the counter electrode and a saturated calomel electrode (Hg/HgCl_2_, SCE) as the reference electrode. We applied a constant voltage (−1 V vs. SCE) and different times (30, 60, and 90 min) for electrochemical deposition to determine the optimal catalyst performance. As a comparison, the corresponding precursor salts can be removed for the synthesis process of NiP and CoP. The electrodeposition time was 60 min. For electrochemical deposition associated with NiCoP catalysts, the specific quality load is provided in Table 1. Specific reagent information, physical characterization, and electrochemical tests are provided in the Supporting Information.

## 3. Results and Discussion

### 3.1. Physicochemical Characterization

**Figure 2:**
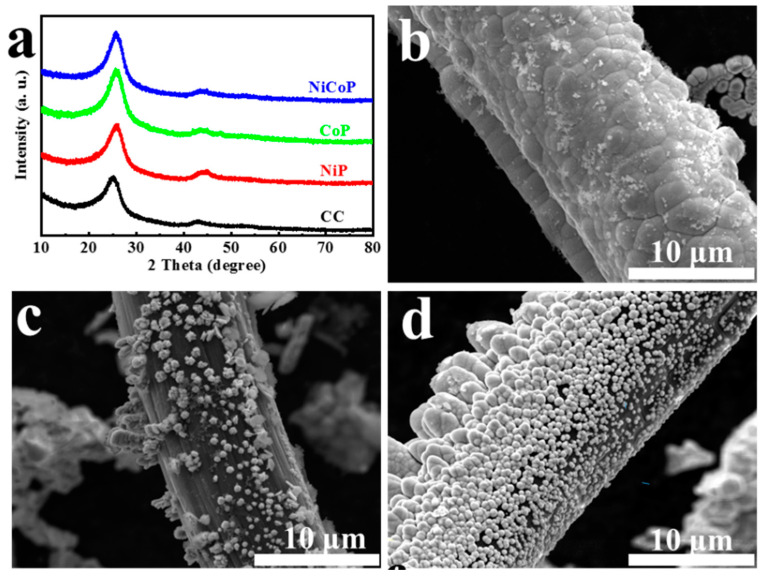
(**a**) XRD of CC, NiP, CoP, and NiCoP. SEM images of (**b**) NiP, (**c**) CoP, and (**d**) NiCoP.

First, XRD characterization (Figure 2a) was used to determine the composition of the phase. Except for the two broad peaks (~25° and 44°) attributed to the conductive substrate carbon cloth, there was no obvious NiCoP diffraction peak in the NiCoP catalyst after electrochemical deposition 1 h. For CoP and NiP samples, the XRD peak shape and peak position were roughly the same as those of NiCoP. The XRD results showed that the phosphating compounds obtained by electrochemistry are amorphous, which was quite different from the samples obtained by phosphides at a high temperature. The morphology of the catalyst is shown in Figure 2b–d and Figures S1–S3, which demonstrated the successful electrochemical growth of relevant phosphides on carbon cloth substrates. Under the same electrodeposition conditions of 1 h, NiP, CoP, and NiCoP particles have a slightly different shape. For NiP, micron-sized spherical particles were bound together and evenly attached to the surface of the CC, and the average size of NiP particles was close to 3 μm. This demonstrated the close contact between the electrochemically deposited NiP and the carbon cloth substrate, implying mechanical stability of the catalyst but a limited contact area with the electrolyte. As for CoP, relatively dispersed nanosheets and stacked flower-like particles were observed in CoP. In addition, large patches of bare and smooth carbon cloth indicated that the catalytically active site of growing active nanocrystals is limited on the surface of CC under the same conditions of electrochemical deposition. Small uniform NiCoP nanoparticles were closely arranged on the CC. As shown in Figures S1–S3, the EDS results displayed that the relevant elements were uniformly distributed in NiP, CoP, and NiCoP, and the atomic ratio (Table S1) of Ni/P was 4:1, that of Co/P was 5.93:1, and that of Ni/Co/P was 2.64:10.64:1.

**Figure 3:**
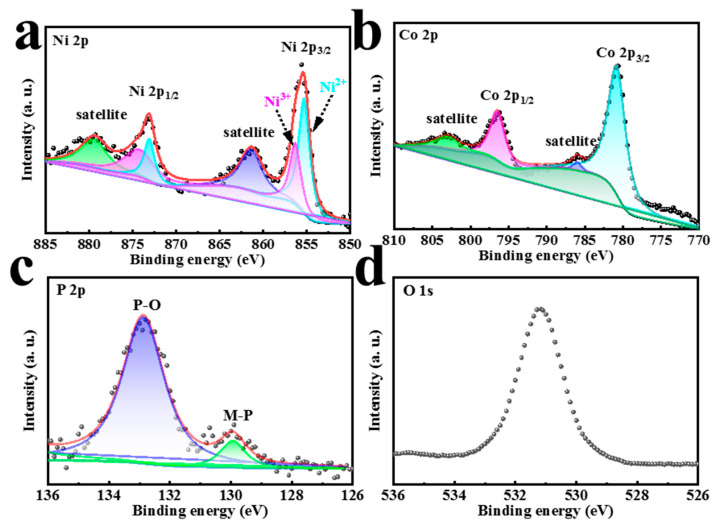
XPS spectra of (**a**) Ni 2p, (**b**) Co 2p, (**c**) P 2p, and (**d**) O 1 s of NiCoP.

X-ray photoelectron spectroscopy (XPS) characterization was also carried out to further illustrate the species information and surface valence of each element in electrochemically deposited phosphating compounds. The survey spectra (Figure S4) of NiCoP showed the existence of Ni, Co, P, and O peaks. For the Ni 2p spectrum (Figure 3a), it can be fitted into six peaks. The peaks of 873.1 eV and 855.3 eV are attributed to Ni^2+^ 2p_1/2_ and Ni^2+^ 2p_3/2_, respectively. Another pair of peak binding energies of 874.4 eV and 856.4 eV belong to Ni^3+^ 2p_1/2_ and Ni^3+^ 2p_3/2_, respectively [28,29]. The final pair of diffraction peaks of 879.6 eV and 861.5 eV are assigned as satellite characteristic peaks of Ni 2p_1/2_ and Ni 2p_3/2_ components, respectively [29]. For the Co 2p spectrum (Figure 3b), there are four decoupled peaks. The binding energies of 780.9 eV and 796.5 eV are certified as Co 2p_3/2_ and Co 2p_1/2_, respectively. Likewise, two shake-up satellite peaks are centered at 803.3 and 786.0 eV [23,30]. The existence of Ni^2+^ and Co^2+^ can accelerate electrolytic water reaction by weakening the O-H bond. Furthermore, the P 2p region (Figure 3c) consists of P-O (132.9 eV) and M-P (129.9 eV) bonds and the O 1s peak (Figure 3d) is centered at 531.2 eV, which suggests the coexistence of the metal phosphides and the oxide species on the NiCoP surface [25,31]. Therefore, the above results just confirm that one-step electrochemical deposition can obtain NiCoP, avoiding the tedious operation steps brought by high-temperature phosphating. A large number of works in the literature have suggested that positively charged Ni^δ +^ /Co^δ+^ and negatively charged P^δ-^ active sites are formed in phosphating compounds, accelerating charge transfer and reducing internal resistance, and thus are favorable to HER and OER activity [28]. Regarding the NiP XPS spectrum (Figure S5), the Ni 2p spectrum can be fitted by the doublet characteristic of Ni^2+^ (855.3 eV and 873.1 eV) and Ni^3+^ (856.5 eV and 874.5 eV) as well as two shake-up satellites (861.6 eV and 879.7 eV). For the CoP sample, there are two shake-up satellite peaks (785.2 eV and 803.3 eV), Co 2p_1/2_ (796.2 eV), and Co 2p_3/2_ (780.2 eV) in the XPS spectrum of Co. In addition, the presence of P elements in both NiP and CoP XPS confirms the formation of mono-metal phosphates.

### 3.2. Electrochemical Measurements

**Figure 4:**
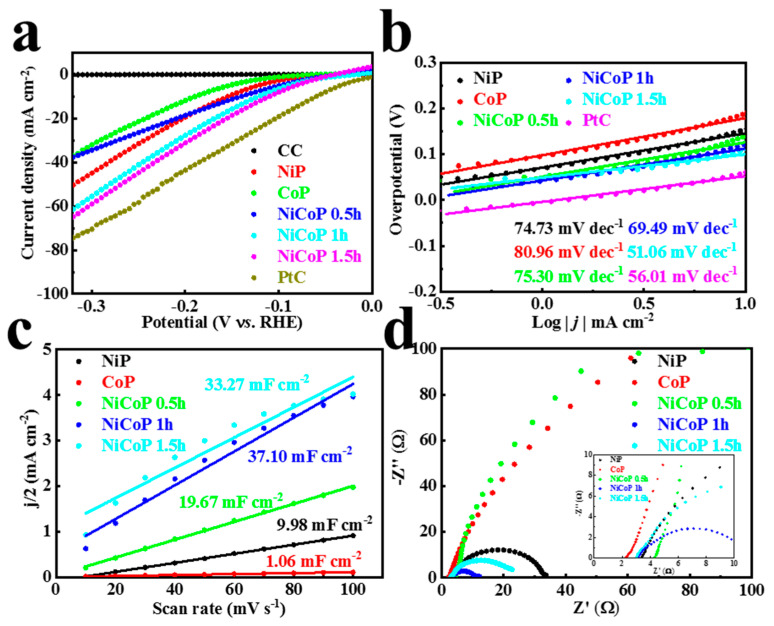
(**a**) LSV curves and (**b**) Tafel plots of CC, NiP, CoP, NiCoP 0.5−1.5 h, and PtC. (**c**) C_dl_ and (**d**) EIS Nyquist plot of NiP, CoP, and NiCoP 0.5−1.5 h in 0.5 M H_2_SO_4_ for HER; the inset presents the plot enlarged over the high frequency range.

Subsequently, HER activities of mono-metal NiP and CoP and bimetallic NiCoP during 1 h of electrochemical deposition were evaluated in an acidic three-electrode system (0.5 M H_2_SO_4_). As shown in Figure 4a, on the LSV curve, except for PtC, NiCoP for 1 h was the optimal HER catalytic activity (as low as 110 mV vs. RHE overpotential at 10 mA cm^−2^), superior to NiP (154 mV vs. RHE) and CoP (189 mV vs. RHE). The electrochemical deposition time also had a significant effect on the performance of the NiCoP catalyst. In order to obtain 10 mA cm^−2^ current density, an overpotential of 140 mV versus RHE needs to be applied for NiCoP for 0.5 h. For 1 h and 1.5 h, there is little difference in their catalytic performance in NiCoP. A sharp increase in current (Figure S6) during the 1 h electrodeposition showed that the precursor transforms into NiCoP. After 1 h, the current density was generally stable because the increase in the thickness of the catalyst layer affected its conductivity instead. Therefore, from the perspective of catalytic activity and time efficiency, 1 h of electrochemical deposition of NiCoP was determined for the follow-up study. Notably, in order to reveal the kinetics of the electrochemical reaction, the Tafel slope of related catalysts was determined and is presented in Figure 4b. The Tafel slopes of NiP, CoP, and NiCoP 1 h were 74.73, 80.96, and 69.49 mV dec^−1^, respectively, which verified the lowest kinetic energy barrier as well as a fast reaction speed in NiCoP, and meant that HER complied with the Volmer–Heyrovsky mechanism. Further, the Tafel slope of NiCoP decreased with the increase in deposition time from 0.5 to 1.5 h. The NiCoP 1.5 h (51.06 mV dec^−1^) sample even showed reaction kinetics similar to that of PtC (56.01 mV dec^−1^). The electrochemically effective surface area (ECSA) represented the number of active sites of electrocatalysts, estimated via the cyclic voltammetry (CV) method (Figure S7). As shown in Figure 4c, the double-layer capacitance (C_dl_) value of NiP, CoP, NiCoP 0.5 h, NiCoP 1 h, and NiCoP 1.5 h was 9.98, 1.06, 19.67, 37.10, and 33.27 mF cm^−2^, respectively, in which the value of the NiCoP 1 h was obviously the largest and manifested more active sites. The electrochemical impedance spectra (EIS) of the catalysts (Figure 4d) in the region of high and low frequencies were then investigated to elucidate charge transfer resistance (R_ct_). It can be seen that the value of fitting R_ct_ of NiCoP 1 h was 8.14 Ω (Table S2) based on an equivalent circuit model (Figure S8), which indicates a faster electron transfer rate. It was much lower than that of NiP (30.19 Ω), CoP (371.00 Ω), NiCoP 0.5 h (326.00 Ω), and NiCoP 1.5 h (20.58 Ω). The above results all confirmed that the bitransition metal phosphide promoted the electrochemical HER catalytic reaction compared with NiP or CoP alone. The electron transfer rate and reactive site of NiCoP can be further regulated by an appropriate electrochemical deposition time.

**Figure 5:**
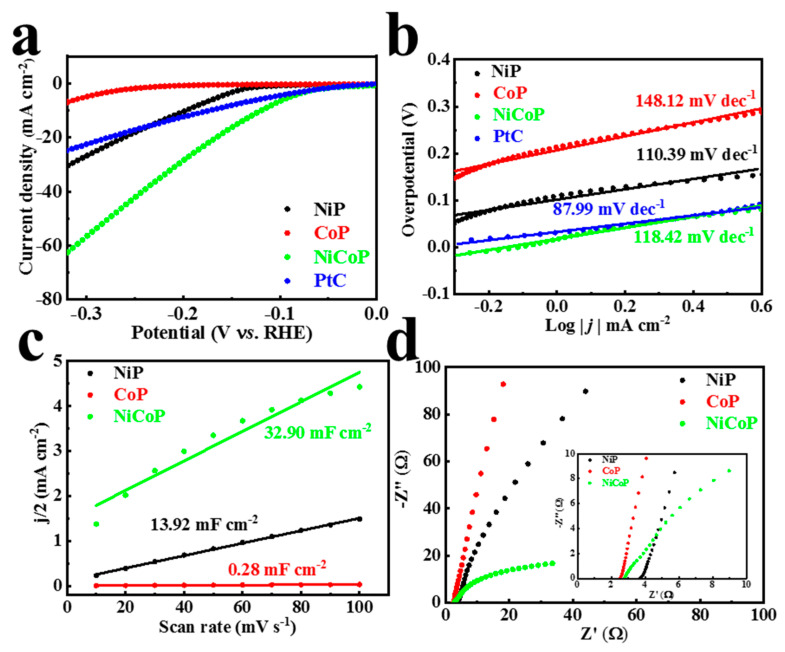
(**a**) LSV curves and (**b**) Tafel plots of NiP, CoP, NiCoP 1 h, and PtC. (**c**) C_dl_ and (**d**) EIS Nyquist plot of NiP, CoP, and NiCoP 1 h in 1 M KOH for HER; the inset presents the plot enlarged over the high frequency range.

Similarly, in order to show the universality of the catalyst in different electrolytes, we also evaluated the HER performance of NiCoP under alkaline conditions of 1 M KOH. Three phosphides, including NiP, CoP, and NiCoP, under the same 1 h deposition time were tested for HER activity (Figure 5a). Similar to acidic conditions, NiCoP showed the best performance of 120 mV overpotential, even better than commercial PtC (173 mV). The Tafel slopes (Figure 5b) for NiP, CoP, and NiCoP are 110.39, 148.12, and 118.42 mV dec^−1^, respectively. These values suggest that the HER of these catalyst proceeds according to the Volmer–Heyrovsky reaction under alkaline conditions. Unsurprisingly, the fitting curve is roughly linear; the highest C_dl_ of 32.90 mF cm^−2^ is still displayed by NiCoP, followed by NiP (13.92 mF cm^−2^) and CoP (0.28 mF cm^−2^) in that order (Figure 5c and Figure S9). According to the R_ct_ value (Figure 5d and Table S3) of the EIS fitting circuit, NiCoP exhibited the lowest R_ct_ of 44.55 Ω among NiP (578.50 Ω) and CoP (2419.00 Ω) monometallic phosphides. The remarkably different low frequency behavior of NiCoP may be attributable to the electrochemical double layer at the surface, whereas the physical origin may be both a single or double Schottky barrier in the substrate–catalyst heterojunction or a hydroxide interface (NiCoP surface reconstruction in an alkaline environment) material created at the boundary [32].

**Figure 6:**
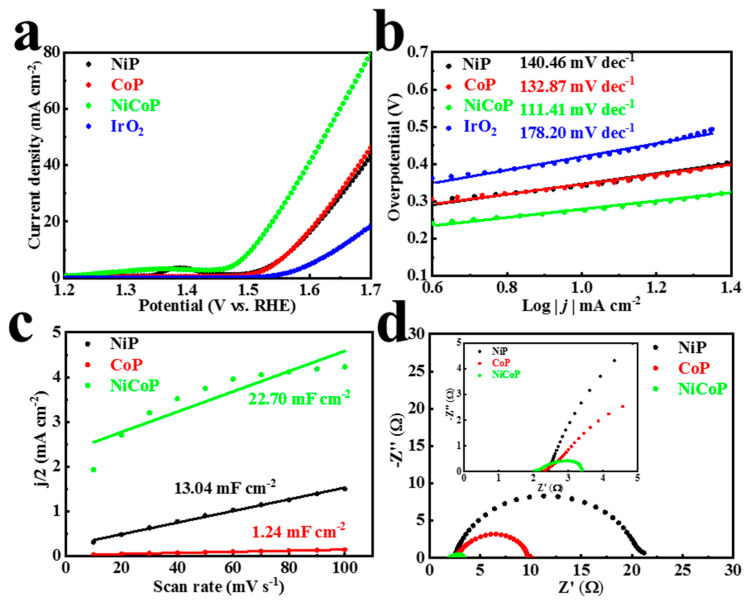
(**a**) LSV curves and (**b**) Tafel plots of NiP, CoP, NiCoP 1 h, and IrO_2_. (**c**) The double−layer capacitance (C_dl_) and (**d**) EIS Nyquist plot of NiP, CoP, and NiCoP 1 h in 1 M KOH for OER; the inset presents the plot enlarged over the high frequency range.

In addition to excellent HER characteristics, the OER characteristics of materials also have an important impact on the final water electrolysis efficiency. Compared with the simple HER reaction mechanism, it is often more difficult to break through the reaction barrier of OER to develop an active catalyst. To illustrate the prospective of one-step electrochemical deposition of NiCoP in water electrolysis, the catalytic activity of OER at 1 M KOH was studied. NiCoP presented an outstanding OER activity. It only required a low overpotential of 276 mV to achieve a current density 10 mA cm^−2^ (Figure 6a), which was superior to those of NiP (342 mV) and CoP (341 mV). The Tafel slopes based on polarization curves are presented in Figure 6b. The Tafel slope for NiP, CoP, and NiCoP was 140.46, 132.87, and 111.41 mV dec^−1^, respectively, which surpassed commercial IrO_2_ (178.20 mV dec^−1^) toward OER. In an alkaline solution, the fitting curve (Figure 6c and Figure S10) is roughly linear. The C_dl_ value of NiCoP is up to 22.70 mF cm^−2^, higher than that of NiP (13.04 mF cm^−2^) and CoP (1.24 mF cm^−2^). Similarly, NiCoP has the fastest electron transfer capability, with R_ct_ as low as 1.43 Ω (Figure 6d and Table S4). All electrochemical data are provided in Tables S5 and S6.

**Figure 7:**
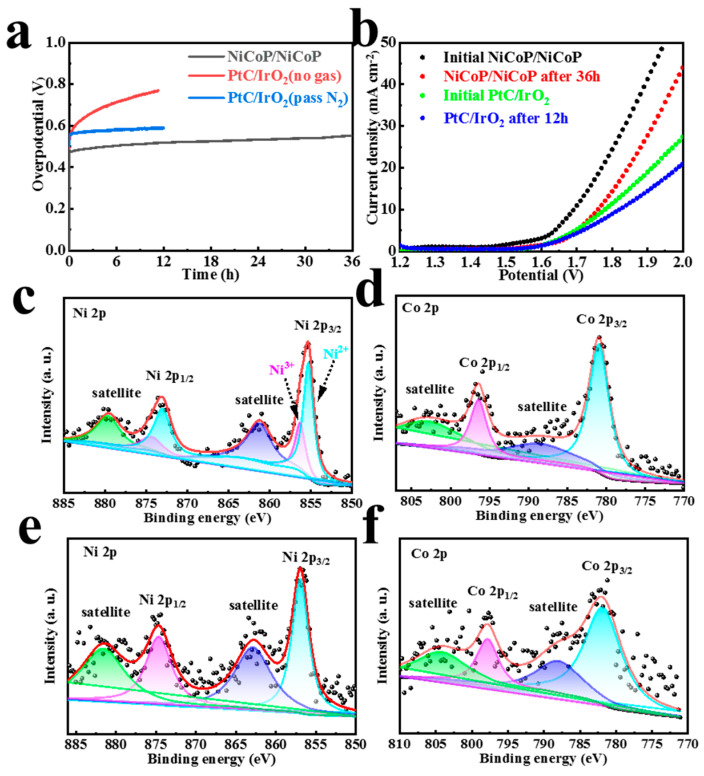
(**a**) Water splitting stability test of NiCoP/NiCoP and PtC/IrO_2_. (**b**) LSV curves of NiCoP/NiCoP and PtC/IrO_2_ before and after overall water splitting. High-resolution XPS spectra of (**c**) Ni 2p and (**d**) Co 2p after HER in 1.0 M KOH. High-resolution XPS spectra of (**e**) Ni 2p and (**f**) Co 2p after OER in 1.0 M KOH.

In view of the excellent HER and OER performance of NiCoP, we assembled an overall water splitting device, in which NiCoP served as both the anode and cathode in 1 M KOH. Impressively, NiCoP/NiCoP afforded current densities of 10 mA cm^−2^ when the cell voltage was 1.69 V (Figure 7), with combined overpotentials of 460 mV. Impressively, symmetrical NiCoP/NiCoP catalysts were superior to the commercial PtC/IrO_2_ couple. It is comparable to the conventional transition metal phosphide reported recently (Table S7). The stability evaluation of catalysts is of great significance for the application of water splitting. As is illustrated in Figure 7a, NiCoP/NiCoP showed superior durability in the overall water splitting reaction with slight overpotential fluctuation at 36 h of continuous electrolysis. In the absence of continuous N_2_ injection to eliminate the electrode bubble operation, the overpotential at 10 mA cm^−2^ current density increased by about 70 mV after a long period of electrolysis. The chronopotentiometry curve clearly indicated that commercial PtC/IrO_2_ catalysts deactivated significantly after 12 h of the stability test as a result of the formation of large bubbles. In order to confirm this speculation, the commercial catalyst can also remain stable during continuous electrolysis when N_2_ gas is introduced continuously to eliminate bubbles. For NiCoP, the phenomenon is less pronounced. This is mainly because of the structure of the catalyst surface and the difference in hydrophilic and hydrophobic properties. Thus, NiCoP still shows an advantage over commercial catalysts, especially in the actual water electrolysis environment. The structural evolution of the tested samples was also revealed by physical characterization in order to understand the transformation of catalyst activity. The XRD (Figure S11a) of NiCoP as cathode after HER durability still revealed no additional obvious phase formation, and the anodic NiCoP also performed almost identically after the OER cycle. According to SEM images in Figure S11b, the NiCoP of the cathode was transformed into an agglomerated nanoflowers morphology. For the anode (Figure S11c), the particles were still spherical, but larger than the original particle size of NiCoP. These results indicate that structural reconstruction of NiCoP may occur, but the performance retention rate has not completely failed during the long-term operation of water splitting. According to the XPS test (Figure 7c–f and Figure S12), we can obtain the change in valence state and structural reconstruction of NiCoP chemicals in the cathode and anode of overall water splitting during a long time period of electrolysis. After HER, there are no obvious peak position changes in the Ni and Co 2p peak of NiCoP cathode (Figure 7c,d). Three groups of Ni 2p (Ni^2 +^: 873.1 eV and 855.4 eV, Ni^3 +^: 874.4 eV and 856.4 eV, satellite: 879.7 eV and 861.2 eV) and Co 2p (Co 2p_1/2_: 796.5 eV, Co 2p_3/2_: 780.9 eV, and satellite: 803.1 eV and 788.4 eV) fitting peaks show that the transition metal element valence state of NiCoP has no change in the process of HER. It is worth noting that the XPS peak of P element (Figure S11b) after HER is almost invisible and the characteristic peak (M-OH and M-O) of O 1s (Figure S11c) located at binding energies of 532.4 eV and 531.0 eV appears. The above results indicate that the surface of NiCoP has been reconstructed to Ni(OH)_2_ or Co(OH)_2_ with P loss of the pristine electrode after the HER test [33]. For the anode of NiCoP, the valence state of Ni and Co has elevated compared with the original sample according to the binding energy (Figure 7e,f) of Ni(Co) 2p. Specifically, the Ni 2p distinct peak at 874.7/857.0 eV (Ni 2p_1/2_/Ni 2p_3/2_) and 881.7/862.9 eV (satellite), which corresponds to NiOOH, underwent a complete positively shift after OER [34,35]. The XPS location of Co 2p also appears to display an obvious positive shift, representing the existing of CoOOH [36,37]. Similarly, the P peak also disappears and the O 1s peaks shift positively to 532.8 eV (M-OH) and 532.0 eV (M-O) (Figure S11d,e). The above results demonstrate that NiCoP can be reconstructed into high-valence NiCo oxyhydroxides by the electrochemical OER process [26]. Thus, the evolution of surface hydroxides or oxyhydroxides can continue to undergo electrocatalytic reactions in spite of the attenuation of water splitting activity because of the absence of P element.

## 4. Conclusions

Highly active phosphides (NiP, CoP, and NiCoP) were obtained through a simple one-step electrodeposition method. Compared with the late high temperature phosphating treatment, a new scheme was added for the preparation of phosphide materials. The effects of electrolyte composition and deposition time on electrocatalytic performance were studied systematically. Through compositional optimization, NiCoP achieved the high catalytic activity for HER in different pH solutions, only requiring an overpotential of 111 mV in 0.5 M H_2_SO_4_ and 120 mV in 1 M KOH at 10 mA cm^−2^. Under alkaline conditions, excellent OER activity was also observed, with an overpotential of 276 mV at 10 mA cm^−2^ without IR correction. The overall water splitting device composed of NiCoP/NiCoP was assembled with an initial cell voltage of 1.69 V to achieve 10 mA cm^−2^ current density in 1.0 M KOH. Subsequently, the surface valence and structural evolution of NiCoP samples with slowly decaying stability under alkaline conditions are revealed. NiCoP is reconstructed into Ni(Co)(OH)_2_ hydroxides (for HER) and Ni(Co)OOH oxyhydroxides (for OER) on the surface with P element loss, acting as real “active sites”.

## Figures and Tables

**Table 1:** The mass load of the relevant catalyst.

Catalysts	Mass Loading (mg cm^−2^)
NiP	1.8 ± 0.2
CoP	1.5 ± 0.2
NiCoP 0.5 h	0.7 ± 0.2
NiCoP 1 h	2.0 ± 0.2
NiCoP 1.5 h	4.2 ± 0.2

## Data Availability

The data presented in this study are available on request from the corresponding author.

## Supplementary Materials

The following supporting information can be downloaded at: https://www.mdpi.com/article/10.3390/ma16041529/s1, Figure S1: SEM and EDS elemental mapping of NiP, Figure S2: SEM and EDS elemental mapping of nanoparticle CoP, Figure S3: SEM and EDS elemental mapping of nanoparticle NiCoP, Figure S4: XPS survey scan spectra of NiCoP, Figure S5: High resolution NiP XPS spectra of (a) Ni 2p and (c) P 2p. High resolution CoP XPS spectra of (b) Co 2p and (d) P 2p, Figure S6: The relationship between current and time during the NiCoP 1.5 h electrodepostion, Figure S7: CV curves of (a) NiP, (b) CoP, (c) NiCoP 0.5 h, (d) NiCoP 1 h, and (e) NiCoP 1.5 h at various scan rates in 0.5 M H_2_SO_4_, Figure S8: An equivalent circuit model based on electrochemical impedance spectrums, Figure S9: CV curves of (a) NiP, (b) CoP, and (c) NiCoP 1 h at various scan rates in 1 M KOH for HER, Figure S10: CV curves of (a) NiP, (b) CoP, and (c) NiCoP 1 h at various scan rates in 1 M KOH for OER, Figure S11: (a) XRD patterns of NiCoP before and after electrocatalytic reaction (HER and OER) in 1 M KOH. SEM images of NiCoP after (b) HER and (c) OER in 1 M KOH, Figure S12: (a) XPS survey spectrum of the NiCoP after water splitting. High resolution XPS spectra of (b) P 2p and (c) O 1s after HER in 1.0 M KOH. High resolution XPS spectra of (d) P 2p and (e) O 1s after OER in 1.0 M KOH, Table S1: Summary of the atomic percentages of NiP, CoP, NiCoP obtained from the EDS spectrum, Table S2: The fitted parameters of the EIS data of NiP, CoP, NiCoP 0.5 h, NiCoP 1.0 h, and NiCoP 1.5 h catalysts for HER in 0.5 M H_2_SO_4_, Table S3: The fitted parameters of the EIS data of NiP, CoP, NiCoP 1 h catalysts for HER in 1.0 M KOH, Table S4: The fitted parameters of the EIS data of NiP, CoP, NiCoP 1 h catalysts for OER in 1.0 M KOH, Table S5: Summary of the HER performance of all electrocatalysts, Table S6: Summary of the OER performance of all electrocatalysts, Table S7: Comparison of the catalytic activity of NiCoP with recently reported non-precious metal electrocatalysts toward the water-splitting in the 1 M KOH. References [38,39,40,41,42,43,44,45,46,47,48] are cited in the Supplementary Material.
