# Supplementary material for: One-Step Electrochemical Synthesis and Surface Reconstruction of NiCoP as an Electrocatalyst for Bifunctional Water Splitting
Source: Materials (Basel). 2023 Feb 11;16(4):1529. doi: 10.3390/ma16041529 (PMC9959249; doi:10.3390/ma16041529)
Supplement: Supplementary file 1 [file materials-16-01529-s001.zip › materials-2192986-supplementary.pdf]

Supporting file

One-Step Electrochemical Synthesis and Surface Reconstruction of NiCoP as an Electrocatalyst for Bifunctional Water Splitting

Minhao Sheng, Yawei Yang *, Xiaoqing Bin and Wenxiu Que *

Electronic Materials Research Laboratory Electronic Materials Research Laboratory, International Center for Dielectric Research, Shaanxi Engineering Research Center of Advanced Energy Materials and Devices, School of Electronic Science and Engineering, Xi'an Jiaotong University, Xi'an 710049, China

* Correspondence: ywyang@xjtu.edu.cn (Y.Y.); wxque@xjtu.edu.cn (W.Q.)

1. Methodology

1.1. Materials:

Cobalt chloride hexahydrate ($CoCl_2·6H_2O$, 98.0%), nickel dichloride hexahydrate ($NiCl_2·6H_2O$, 98.0%), sodium hypophosphite monohydrate ($NaH_2PO_2·H_2O$, 98.0%), and ethyl alcohol (C_2H_5OH, 99.5%) were purchased from Sinopharm Chemical Reagent Co., Ltd. (China). Acetone was purchased from Xi'an Baoan Chemical Co., Ltd. (China). The PtC (20 wt%, RHΛWN) catalyst was obtained from Jiangsu Aikang Biomedical Research and Development Co., Ltd. (China). IrO_2 (99.9% metals basis, Ir ≥84.5%) was purchased from Shanghai Yuanye Biotechnology Co., Ltd. (China). All chemical reagents were directly used as purchased without further treatment. Carbon cloth (CC) was purchased from Suzhou Sinero Technology Co., Ltd, China (W0S1002) with a thickness of 0.33 mm.

1.2. Physical Characterizations

Micromorphology of the sample was was imaged by a scanning electron microscopy (SEM, JSM6390, JEOL, Japan). Crystal structure of the samples was carried out in an X-ray diffraction diffractometer (XRD, D/max 2200, Rigaku, Japan) with a Cu Kα radiation source (λ = 0.154 nm, 40 kV, 10 mA). The XPS spectra of the samples was obtained by an X-ray photoelectron spectroscopy (XPS, ESCALAB 250, VG Thermo, USA) using Al Kα radiation. All the XPS peaks were calibrated to the C 1 s (284.8 eV) peak.

1.3. Electrochemical measurements:

All electrochemical data tests were performed on the CHI660E electrochemical workstation (Chenhua Instruments Co. Ltd, Shanghai). In a typical three-electrode system using 0.5 M H_2SO_4 (pH = 0.6) or 1.0 M KOH (pH = 14) as electrolyte, we employed CC loaded with above active catalytic materials as working electrode, in which carbon rod electrode was selected as the counter electrode, SCE as reference electrode in 0.5 M H_2SO_4 electrolyte, and Hg/HgO as reference electrode in 1.0 M KOH electrolyte. The recorded voltage value relative to counter electrodes need to be converted to reversible hydrogen electrodes (RHE). According to Nernst equation: E vs. RHE (V) = E vs. SCE (V) + 0.05916 × pH + 0.244 (V) (pH =0.6), E vs. RHE (V) = E vs. Hg/HgO (V) + 0.05916 × pH + 0.098 (V) (pH = 14). All overpotential was not corrected by iR. For HER and OER test, the scan rate of the linear sweep voltammetry (LSV) is set to 5.0 mV s^{-1}. The double layer capacitance (C_{dl}) of the catalytic active material was recorded to revel the electrochemically active surface area by cyclic voltammetry (CV) at various scanning rates (10~100 mV s^{-1}) in the non-Faradaic potential range. Electrochemical impedance spectroscopy (EIS) was measured by applying an AC voltage with an amplitude of 5 mV using a frequency range from 100 kHz~20 mHz. Overall water splitting tests were performed in a two electrode cell system, in which NiCoP served as both anode and cathode in 1 M KOH. The chronoamperometry method (a static current density of 10 mA cm^{-2}) was used to imply stability of the catalyst. Before

measurement, the electrolyte was purged with high-purity N_2 for at least 30 min. No gas was passed in during the test. For comparison, the slurry preparation method for commercial catalysts (PtC and IrO_2 serve as a benchmark) is as follows. Typically, the mixture is composed of 10 mg catalysts, 800 μL ethanol, 185 μL deoxidized ultra-pure water, and 15 μL 5% Nafion. The mixture was then magnetically stirred and ultrasonic for several hours to make it even. The catalytic electrodes were fabricated by drop-casting of the slurry onto CC. Let it dry naturally to ensure that the mass load is about 2 mg/cm^2.

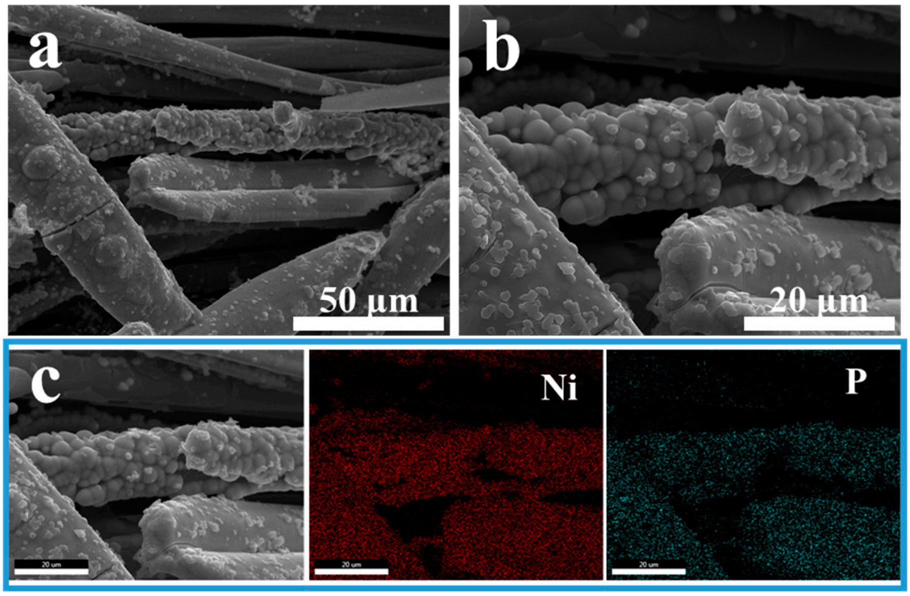

Figure S1. SEM and EDS elemental mapping of NiP.

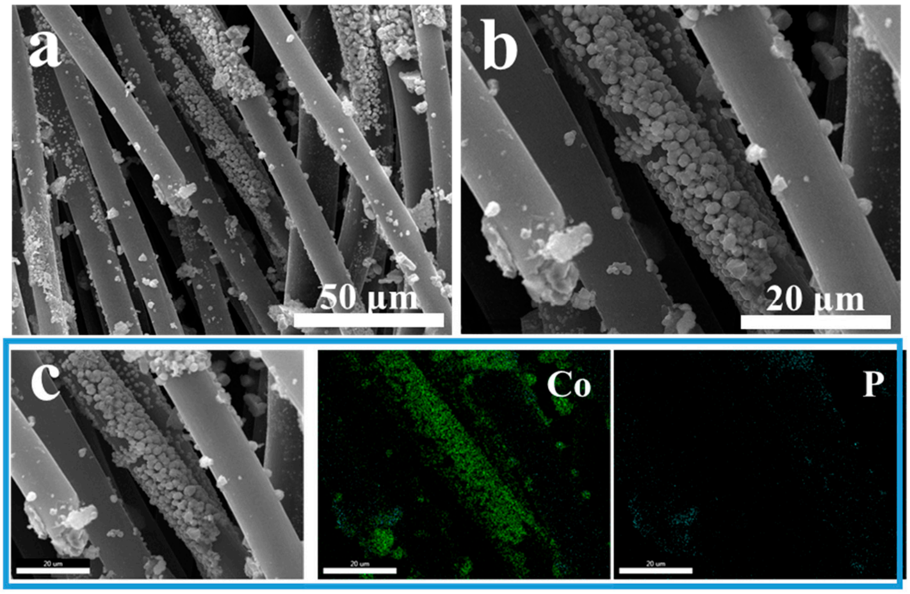

Figure S2. SEM and EDS elemental mapping of nanoparticle CoP.

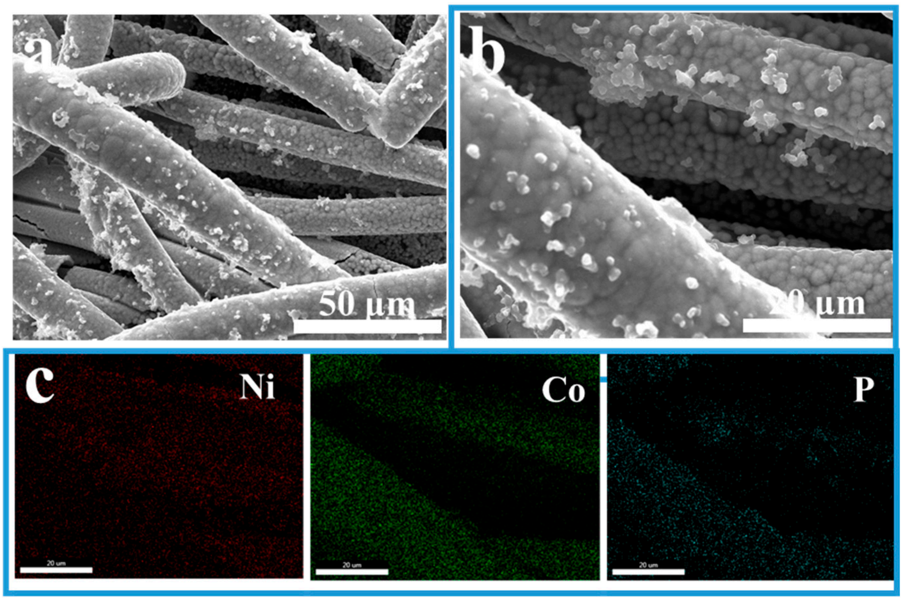

Figure S3. SEM and EDS elemental mapping of nanoparticle NiCoP.

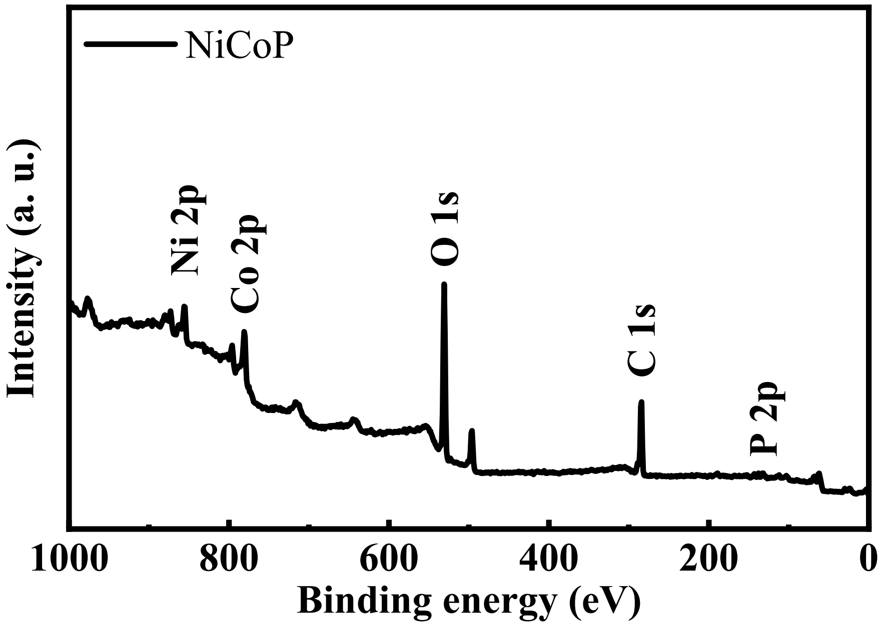

Figure S4. XPS survey scan spectra of NiCoP.

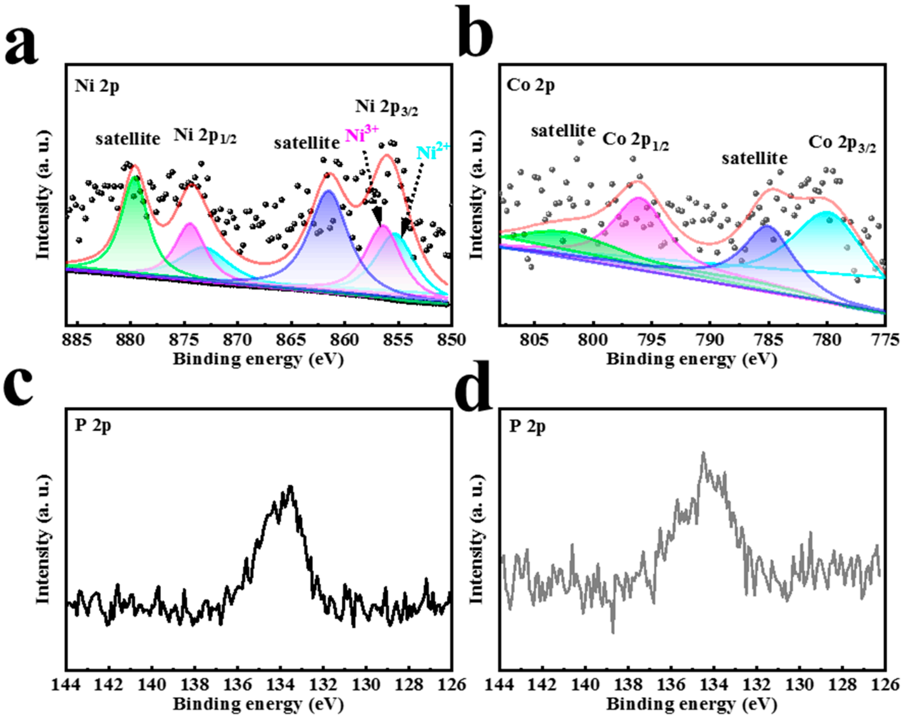

Figure S5. High resolution NiP XPS spectra of (a) Ni 2p and (c) P 2p. High resolution CoP XPS spectra of (b) Co 2p and (d) P 2p.

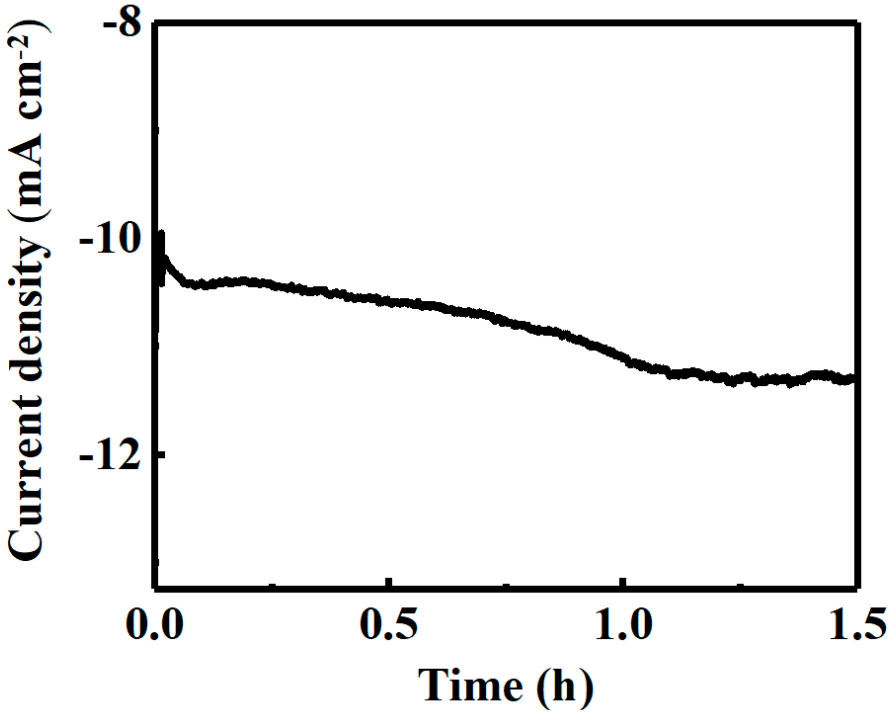

Figure S6. The relationship between current and time during the NiCoP 1.5 h electrodepostion.

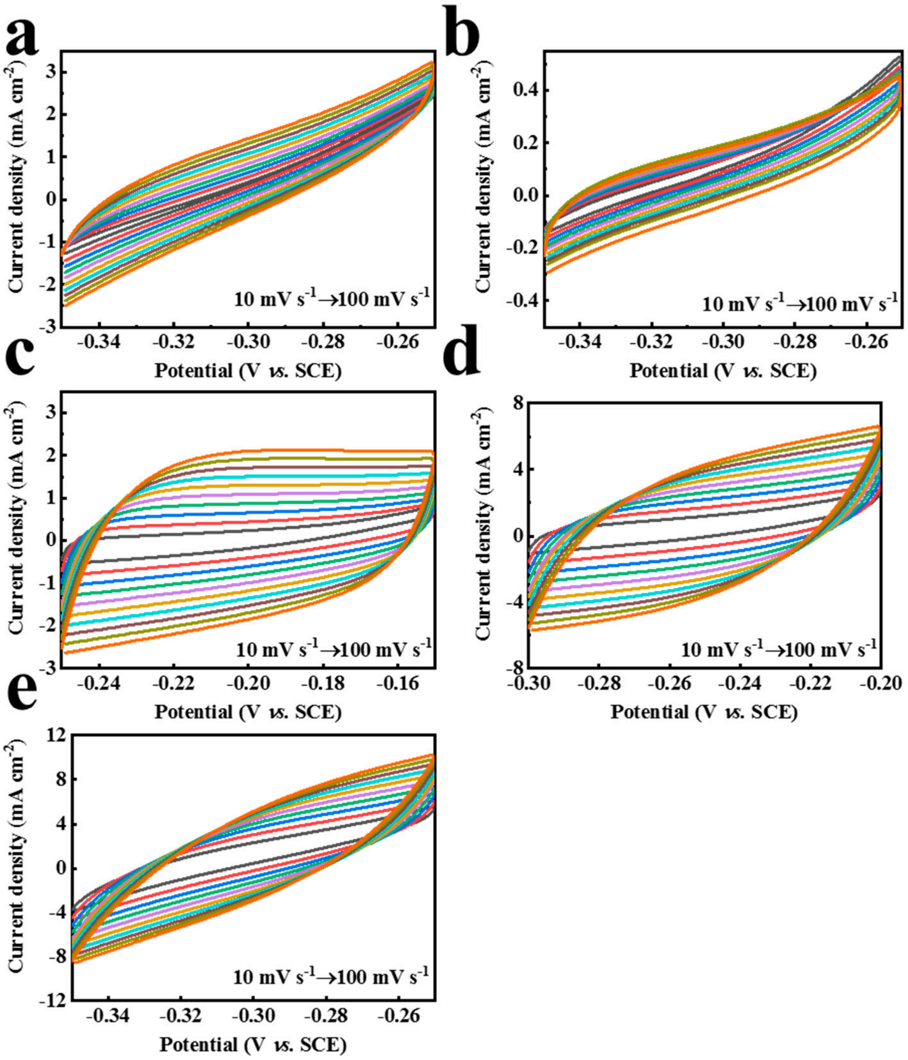

Figure S7. CV curves of (a) NiP, (b) CoP, (c) NiCoP 0.5 h, (d) NiCoP 1 h, and (e) NiCoP 1.5 h at various scan rates in 0.5 M H_2SO_4.

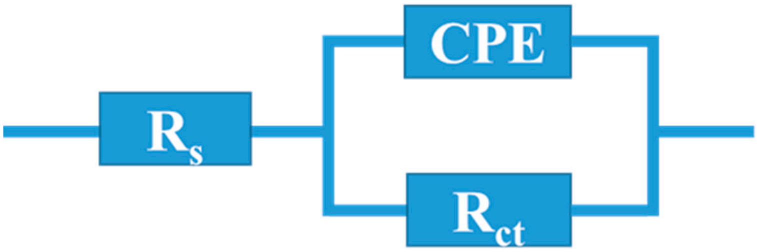

Figure S8. An equivalent circuit model based on electrochemical impedance spectrums.

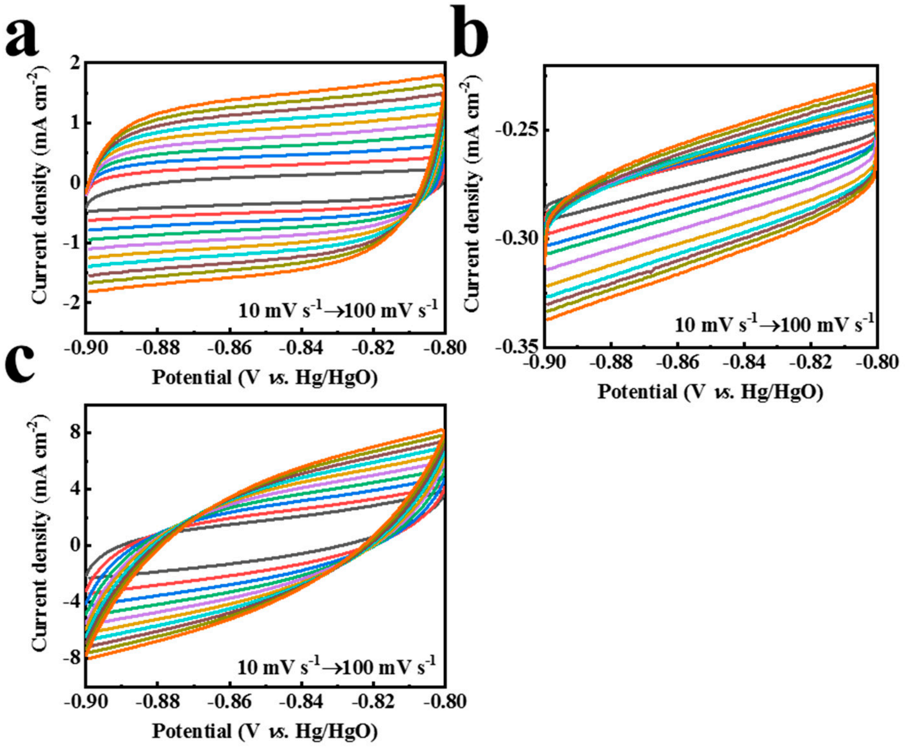

Figure S9. CV curves of (a) NiP, (b) CoP, and (c) NiCoP 1 h at various scan rates in 1 M KOH for HER.

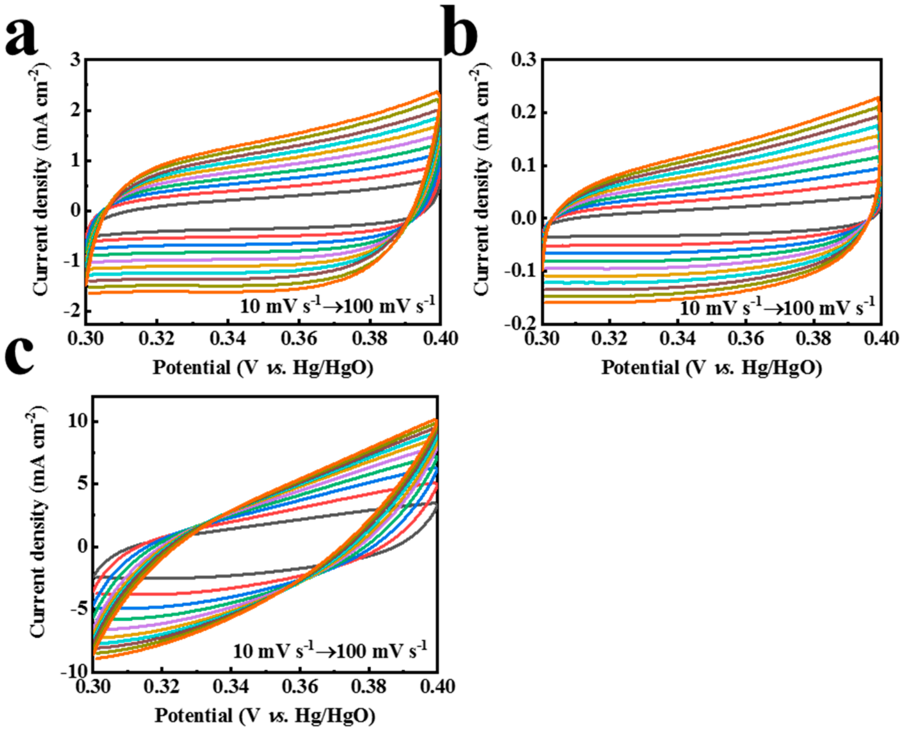

Figure S10. CV curves of (a) NiP, (b) CoP, and (c) NiCoP 1 h at various scan rates in 1 M KOH for OER.

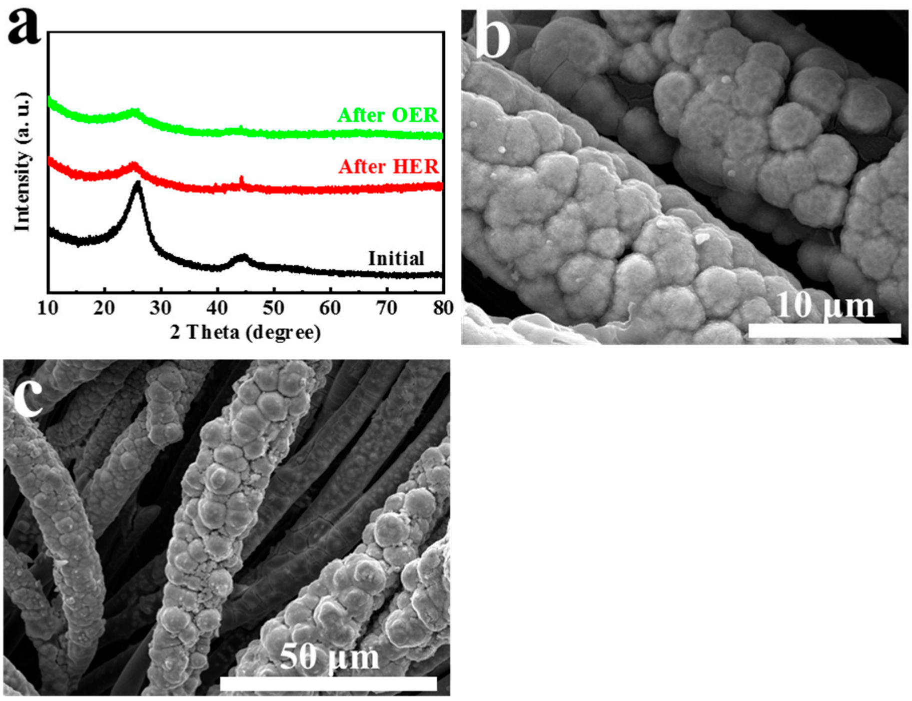

Figure S11. (a) XRD patterns of NiCoP before and after electrocatalytic reaction (HER and OER) in 1 M KOH. SEM images of NiCoP after (b) HER and (c) OER in 1 M KOH.

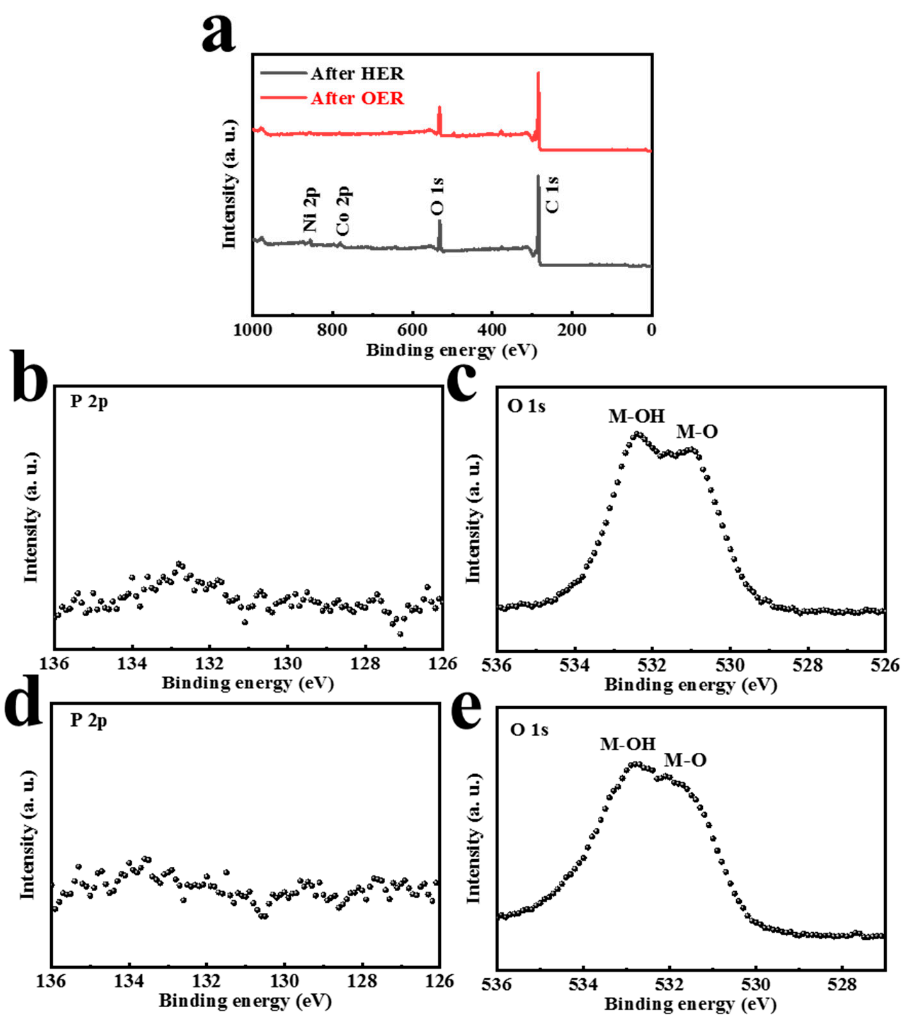

Figure S12. (a) XPS survey spectrum of the NiCoP after water splitting. High resolution XPS spectra of (b) P 2p and (c) O 1s after HER in 1.0 M KOH. High resolution XPS spectra of (d) P 2p and (e) O 1s after OER in 1.0 M KOH.

Table S1. Summary of the atomic percentages of NiP, CoP, NiCoP obtained from the EDS spectrum.

Sample	Ni (at.%)	Co (at.%)	P (at.%)
**NiP**	**80.00**	**--**	**20.00**
**CoP**	**--**	**85.56**	**14.44**
**NiCoP**	**18.50**	**74.50**	**7.0**

Table S2. The fitted parameters of the EIS data of NiP, CoP, NiCoP 0.5 h, NiCoP 1.0 h, and NiCoP 1.5 h catalysts for HER in 0.5 M H_2SO_4.

Catalysts	R_s (Ω)	R_{ct} (Ω)	CPE1-T	CPE1-P
**NiP**	3.409	30.19	0.013468	0.84574
**CoP**	2.418	371.00	0.0012431	0.83755
**NiCoP 0.5 h**	4.339	326.00	0.023060	0.88909
**NiCoP 1.0 h**	3.127	8.14	0.084145	0.78529
**NiCoP 1.5 h**	3.039	20.58	0.10463	0.81882

Table S3. The fitted parameters of the EIS data of NiP, CoP, NiCoP 1 h catalysts for HER in 1.0 M KOH.

Catalysts	R_s (Ω)	R_{ct} (Ω)	CPE1-T	CPE1-P
**NiP**	3.758	578.50	0.016127	0.84316
**CoP**	2.629	2419.00	0.00033888	0.91497
**NiCoP 1 h**	2.730	44.55	0.088210	0.72773

Table S4. The fitted parameters of the EIS data of NiP, CoP, NiCoP 1 h catalysts for OER in 1.0 M KOH.

Catalysts	R_s (Ω)	R_{ct} (Ω)	CPE1-T	CPE1-P
**NiP**	2.387	18.68	0.013072	0.90061
**CoP**	2.387	7.727	0.028328	0.80504
**NiCoP 1 h**	2.068	1.430	0.13419	0.64137

Table S5. Summary of the HER performance of all electrocatalysts.

Catalyst	Electrolyte	η_{10} (mV)	Tafel slope (mV dec^{-1})	C_{dl} (mF cm^{-2})
NiP	0.5 M H_2SO_4	154	74.73	9.98
CoP	0.5 M H_2SO_4	189	80.96	1.06
NiCoP 0.5 h	0.5 M H_2SO_4	140	75.30	19.67
NiCoP 1 h	0.5 M H_2SO_4	110	69.49	37.10
NiCoP 1.5 h	0.5 M H_2SO_4	110	51.06	33.27
NiP	1 M KOH	194	110.39	13.92
CoP	1 M KOH	--	148.12	0.28
NiCoP	1 M KOH	120	118.42	32.90

Table S6. Summary of the OER performance of all electrocatalysts.

Catalyst	Electrolyte	η_{10} (mV)	Tafel slope (mV dec^{-1})	C_{dl} (mF cm^{-2})
NiP	1 M KOH	342	140.46	13.04
CoP	1 M KOH	341	132.87	1.24
NiCoP	1 M KOH	276	111.41	22.70

Table S7. Comparison of the catalytic activity of NiCoP with recently reported non-precious metal electrocatalysts toward the water-splitting in the 1 M KOH.

Catalyst	Current density (mA/cm^2)	η_{HER} (mV)	η_{OER} (mV)	Cell voltage (V)	Ref.
NiCoP	10	120	276	1.69	This work
CoP NWs/CC	100	180	--	--	[38]
NiCoP-NWAs/NF	100	197	--	--	[39]
$(Ni_{0.33}Fe_{0.67})_2P$/NF	50	214	--	--	[40]
NiCoP/CoP/Ti_4O_7	10	128	--	--	[41]
NiFeLDH@NiCoP/NF	10	120	--	--	[42]
NiCoP arrays/NF	10		268		[43]
CoP/CoP_2	10	300	--	--	[44]
NiCoP/C	10	330	--	--	[30]
Co_3S_4 || Ni-Co_3S_4	10	199	283	1.70	[45]
$Ni_{0.9}Co_{0.1}P$/NNC	10	51	221	1.53	[46]
CoP/NF || Co_2P/NF	10	62	280	1.56	[47]
NiS–$Ni_2P_2S_6$/NF	10	140	220	1.64	[48]
